# Insulin resistance (HOMA-IR) and body fat (%) are associated to low intake of fruit and vegetables in Swedish, young adults: the cross-sectional lifestyle, biomarkers and atherosclerosis study

**DOI:** 10.1186/s40795-019-0279-6

**Published:** 2019-02-20

**Authors:** Maria Fernström, Ulrika Fernberg, Anita Hurtig-Wennlöf

**Affiliations:** 1 Åstrand Laboratory of Work Physiology, The Swedish school of sport and health science, GIH, Lidingövägen, 1, 114 86 Stockholm, Sweden; 2 School of Medical Sciences, Faculty of Medicine and Health, Örebro University, 701 82 Örebro, Sweden; 3 School of Health Sciences, Faculty of Medicine and Health, Örebro University, 701 82 Örebro, Sweden

**Keywords:** Body composition, BMI, Body fat (%), Waist circumference, Insulin resistance, HOMA-IR, And dietary habits

## Abstract

**Background:**

In the cross-sectional Lifestyle, Biomarkers, and Atherosclerosis study (LBA study) we have previously reported a high prevalence (15%) of homeostasis model assessment of insulin resistance (HOMA-IR) in Swedish, young adults. The aim of the present study was to report the dietary habits of subjects 18.0–25.9 years, and to associate dietary habits to body composition measures; body mass index (BMI), body fat (%), waist circumference and to HOMA-IR, a risk marker for diabetes.

**Method:**

The subjects (577 women and 257 men) filled in a validated computerized food frequency questionnaire. The questionnaire was based on recommendations from the Swedish national food administration. To associate the dietary habits to BMI, body fat (%), waist circumference and to HOMA-IR the subjects were divided in two groups. Subjects “eating as recommended” and subjects “eating less/more than recommended”.

**Results:**

Recommended intake of fish and seafood (*P* < 0.05), fruit and vegetables (*P* < 0.001), and sweets (*P* < 0.05) were associated to lower HOMA-IR values compared to subjects not eating as recommended. When split by sex no difference in HOMA-IR was detected with recommended intake of fish and seafood, but women eating fish and seafood as recommended had less body fat (%) (*P* < 0.05) compared to women not eating fish and seafood as recommended. Recommended intake of fruit and vegetables was associated to lower HOMA-IR in women (*P* < 0.01), and in women and men to less body fat (%) (*P* < 0.05) compared to subjects not eating the recommended 500 g of fruit and vegetables per day. Both women and men with higher consumption of sweets than recommended had higher HOMA-IR (*P* < 0.05), but no difference in the body composition measures BMI, body fat (%) or waist circumference compared to subjects eating sweets as recommended.

**Conclusion:**

The results highlight the importance of reducing a high intake of sweets and to increase the intake of fish, fruit and vegetables, in young adults, to reduce the risk of future diabetes.

## Background

Despite knowledge and recommendations regarding dietary habits and physical activity, obesity and obesity related diseases, such as diabetes and cardiovascular diseases (CVD), continue to increase [1–3]. Focus on diets and new dietary trends aiming to prevent obesity have led to changes in dietary habits, in Sweden [4]. Dietary and physical activity habits are established in the young adulthood and needs to be evaluated. We have therefore in the Lifestyle, Biomarkers and Atherosclerosis (LBA) study examined 834 young self-reported healthy, non-smoking, Swedish subjects 18.0–25.9 years to survey lifestyle habits and early risk factors for lifestyle related diseases.

In the LBA study risk factors such as blood pressure, triglycerides, high-density lipoprotein cholesterol (HDL-C), glucose, homeostasis model assessment of insulin resistance (HOMA-IR) and high-sensitive C-reactive protein (hs-CRP) were evaluated together to create a risk score. There is no standard for categorizing risk among young adults so we used the definition recommended by Wildman to create the risk score [5, 6]. It was shown that 12% of the subjects, participating in the study, were classified as being at risk to develop CVD in the future [7].

Furthermore the prevalence of insulin resistance (HOMA-IR) was high among the participants in the LBA study. By using the cutoff value > 2.52 for HOMA-IR [5], 15% of the subjects were classified as insulin resistant already before the age of 26 years. Classical risk factors for insulin resistance and diabetes are overweight, abdominal obesity, physical inactivity, smoking and unhealthy dietary habits [8–11]. None of the subjects were smokers and as expected we observed that aerobic fitness was associated with reduced risk to develop CVD in the future. We also observed that subjects with high aerobic fitness had lower HOMA-IR as compared to less well-trained subjects participating in the study [7].

There are several studies on the relation between dietary habits and metabolic risk markers in general but few studies on young adults. Fernandes et al., however, have studied the impact of physical activity choices and healthy dietary habits on coronary heart disease (CHD) risk factors in college students, 18–24 years of age, in the USA. They found that dietary habits and BMI are better indicators of CHD risk as compared to physical activity choices in this age group [12]. Since diabetes is rapidly becoming one of the main health issues and lifestyle changes are the most effective way of preventing or delaying the development of type 2 diabetes [13] it is of importance to study the relation of dietary habits, body composition and HOMA-IR in young adults.

All participants in the LBA study filled in a validated food frequency questionnaire “Matvanekollen” [14]. The result was presented automatically after the test to the participants as a score from 1 to 12 points and have previously been published [7]. The test was based on dietary recommendations from the Swedish national food administration [15], and included questions on seven diet groups. For details see method section.

The aim of the present study was to report the dietary habits of young Swedish adults, by analyzing the result from the seven food groups that made up the food frequency questionnaire. Further on divide the result in groups, subjects “eating as recommended” and subjects “eating less/more than recommended”.

Additionally the aim was to associate the dietary habits to metabolic risk markers; BMI, body fat (%), waist circumference, and insulin resistance (HOMA-IR), a typical risk marker for diabetes.

## Methods

### Study population

In total 834 subjects were included in the cross-sectional study, 577 women and 257 men. To be included the subjects should be 18.0–25.9 years old, healthy and non-smokers. Recruitment was done by advertisement in social media, local newspaper and at the University web platform. The study sample consisted mostly of students from Örebro University. The ethics committee of Uppsala approved the study design, Dnr: 2014/224. All subjects gave their written consent to participate and were informed that they could terminate their participation at any time.

The design of the LBA study has previously been reported in detail [7].

### Questionnaires

All subjects filled in a validated computerized questionnaire about their general physical and mental health [16]. In addition, there were questions about diet and exercise habits. The subjects also stated whether they were vegetarians or following any other specific diet.

Furthermore, they filled in a validated computerized food frequency questionnaire, “Matvanekollen”, on dietary choices [14]. The test was based on recommendations from the Swedish national food administration [15]. The questions are related to the dietary recommendations, and categorized into seven groups based on: 1. Bread (whole grain) 2. Fish and seafood 3. Fruit and vegetables 4. Fat 5. Cheese 6. Sweets (candy, buns, soft drinks, and French fries) 7. Fast food including sausage.

The questions about bread are related to the intake of whole grain. To reach the recommended intake bread with whole grain should be eaten three times per day or more. Fish or seafood should be eaten as main course two to three times per week or more. To reach the recommended 500 g of fruit and vegetables per day, fruit and vegetables should be eaten five times per day or more. Questions about fat, cheese and fast food are related to the total intake of fat and type off fat. Unsaturated fat should preferably be chosen on sandwich and in cooking. The intake of cheese should be restricted and fast food or sausages eaten as main course once a week or less. Candy, buns and soft drinks constitute the group of “sweets”, to which also French fries are included [15]. In this context the group sweets including French fries reflects the content of sucrose. Sweets should be eaten less than three times per week.

The previously published test score [7] was based on the total response of the questionnaire and presented automatically after the test to the participants as a score from 1 to 12 points. In connection, the participants also received advices on how to improve their diet.

In the present study the result from each of the seven food groups have been analyzed separately based on the recommended frequencies for each of the food groups and divided in two groups. Subjects “eating as recommended” and subjects “eating less/more than recommended”.

The analyze was based on a report on indicators for healthy food choices, also from the Swedish national food administration [17]. For example, to eat fish and seafood three times a week or more means that the subject reached the recommended intake of fish and seafood, and to eat fruit and vegetables 5 times a day or more means that the subject reached the recommended intake of fruit and vegetables. The national food administration is commissioned by the government in Sweden to assess the eating habits of the population [17].

### Body composition

Height was measured with a fixed stadiometer to the nearest 0.5 cm, with the subjects standing without shoes, heels together, back straight, and arms extended alongside the body. Waist circumference was measured around the abdomen between iliac crest, and the lowest rib on exhalation, to the nearest 0.5 cm using a flexible, non-stretchable, measuring tape [18].

Body weight, body mass index (BMI) and percentage body fat were measured on Tanita, a bioelectrical impedance body composition analyzer, (Tanita Europe B.V. Tanita BC-418 MA, Amsterdam, Nederland’s). The procedure was performed with the subjects standing barefoot on the metal surface conductive-equipment according to the manufacturer’s guidelines. Adjustments were made with 1 kg for clothes and the standard setting was used [19].

### Measurement of metabolic biomarkers

Blood samples were collected in the morning following an 8–12 h fasting period. The venipuncture was performed with a 21 gauge butterfly needle (Greiner Bio-One International GmbH, Vacuette®, Rainbach im Mühlkreis, Austria. After blood collection all tubes (BD AB, BD Vacutainer, Stockholm, Sweden) were gently inverted several times.

One 3 mL citric acid-citrate-NaF tube was used to collect blood to analyze glucose. After extended inverting of the tube to prevent hemolysis it was placed in room temperature until transportation to the accredited clinical chemistry laboratory at Örebro University Hospital.

Serum to analyze insulin was obtained by collecting blood in a 4 mL standard serum tube with clot activating substances. Before centrifugation at 2000×g for 8 min in room temperature the blood was allowed to clot for at least 30 min before transportation to the clinical chemistry laboratory at Örebro University Hospital. Insulin (mU/L) was analyzed on an Architect i2000SR instrument from Abbott (Illinois, U.S.A.), with their reagent according to their instructions on antibody-based technologies.

### Insulin resistance

HOMA-IR was calculated by using the mathematical equation from Matthew, (insulin (mU/ml)*glucose (mmol/L)/22.5), and the ratio HOMA-IR was used as a measure of insulin resistance [20].

### Statistical analyses

The nominal food frequency questionnaire data was classified into two groups, the subjects reaching the recommendations according to the National food administration [17] were assigned into the group “eating as recommended” and the rest of the subjects were classified as “eating less/more than recommended”.

Analyses were performed both non-gender specific and gender specific. Variables were checked for normal distribution before statistical analyses. Differences in body composition measures and HOMA-IR between the two groups (i.e. “eating as recommended” versus “eating less/more than recommended”) were tested by unpaired Student’s t-test or by a two way ANOVA followed by a post hoc test. Level of significance was set to *p* < 0.05.

Statistical calculations were performed using IBM SPSS Statistics, version 24.0 for Windows (IBM Corp, Armonk, New York, USA).

## Results

Of the 840 subjects who volunteered to participate 6 subjects were excluded. All remaining 834 subjects filled in the food frequency questionnaire. In total, 10% of the subjects reported being born outside Sweden and 24% of the subjects reported having at least one parent born outside Sweden. Questions about the type of lipid used on sandwiches, saturated or unsaturated, was answered by 813 subjects, the remaining 21 subjects answered that they did not know. Due to drop out or technical difficulties, there are some missing values on body composition and metabolic biomarkers. Results for waist circumference was reported in 833 subjects: glucose in 821 subjects and insulin in 815 subjects. HOMA-IR was calculated for 809 subjects. The basic characteristics of the subjects are presented in Table 1.

**Table 1:** Basic characteristics of *n* = 834 subjects divided by sex, 577 women and 257 men, data are presented as mean and standard deviation (STD) or as median^a^ and interquartile range (Q1-Q3)

	Women	Men
Age (year)	21.8 (1.9)	22.0 (2.0)
BMI (kg/m^2^)	22.4 (3.6)	23.4 (3.1)
Body fat (%)	28.0 (6.6)	14.8 (5.6)
Waist circumference (cm)	73.9 (7.9)	81.9 (7.3)
Glucose (mmol/L)	4.9 (0.3)	5.2 (0.3)
Insulin (mU/L)	8.0 (4.5)	7.5 (3.7)
HOMA-IR	1.8 (1.1)	1.8 (0.9)
Food habits (points)^a^	6 (5–7)	6 (4–7)

*Abbreviations: BMI* = body mass index and HOMA-IR = homeostasis model assessment of insulin resistance

Among the subjects 6.3% were vegetarians and 5.9% stated that they were follow a low-carbohydrate-high–fat (LCHF) like diet.

The adherence to the recommendations from the Swedish national food administration [15] regarding selected food categories are presented in Table 2.

**Table 2:** Adherence to the recommendations from the Swedish national food administration [15], regarding the seven food groups, total *n* = 834 except for group number 4, total *n* = 813

Group	Food type	Follows the recommendation (%)
1	Bread	11.4%
2	Fish and seafood	11.4%
3	Fruit and vegetables	15.5%
4	Fat	30.6%
5	Cheese (20–40%)	37.7%
6	Sweets	40.9%
7	Fast food	89.2%

Clarification: To reach the recommendations the subjects should; eat bread with whole grain three times per day or more, eat fish and seafood two to three times per week or more, and eat fruit and vegetables five times per day or more. Preferably chose unsaturated fat and eat cheese and sausages once a week or less. The group named “sweets” includes candy, buns, soft drinks and French fries. Candy, buns, soft drinks, and French fries should be eaten less than three times a week

In the present study 88.6% of the participants did not reach the recommended intake of bread (whole grain) or fish and seafood, 84.5% of the participants did not reach the recommended intake of fruit and vegetables and 59.1% of the young Swedish adults had a higher than recommended intake of candy, buns, soft drinks and French fries.

The questionnaire contained questions about food choices rich in saturated fat. On the question about the intake of cheese (20–40%), 62.3% answered that they eat cheese more often than recommended. On questions about the type of fat used, 23% of the subjects stated that they typically use saturated, and 77% that they use unsaturated fat in cooking. 52% of the subjects stated that they usually choose saturated fat on sandwiches, 11% did not use fat on sandwiches, and the remaining 37% choose unsaturated solid fat on sandwiches. The majority of the subjects, 89.2%, were following the recommendations on fast food and sausages.

### Body composition

When fish and seafood consumption were associated to body composition measures (i.e. BMI, body fat % and waist circumference) it was shown that women eating fish and seafood according to the recommendations had significantly less body fat (%) (*P* < 0.05), but no difference in BMI or waist circumference as compared to women not eating fish or seafood as main course twice a week or more often. In men no association to body composition measures were observed with the recommended intake of fish and seafood.

When fruit and vegetable consumption were associated to the body composition measures it was shown in both women and men that subjects reaching the recommended intake of fruit and vegetables had significantly less body fat (%), women (*P* < 0.05) and men (P < 0.05), but no difference in BMI or waist circumference.

Women and men with high consumption of sweets i.e. candy, buns, soft drinks and French fries had no difference in BMI, body fat (%) or waist circumference as compared to subjects eating sweets as recommended, see Table 3.

**Table 3:** The table shows Body fat (%) values for the groups “eating as recommended” and “not eating as recommended” divided by sex, for three of the seven food groups. Significance level was set to *P* < 0.05, n.s. = not significant

Group	Food type	Body fat (%)	Body fat (%)	*P* value
		Women eating as recommended	Women not eating as recommended	
2	Fish and seafood	26.4	28.3	P < 0.05
3	Fruit and vegetables	26.5	28.4	P < 0.05
6	Sweets	28.0	28.1	n.s.
		Men eating as recommended	Men not eating as recommended	
2	Fish and seafood	14.2	14.9	n.s.
3	Fruit and vegetables	12.4	15.1	P < 0.05
6	Sweets	15.0	14.7	n.s.

Clarification: The group named “sweets” includes candy, buns, soft drinks and French fries

All body composition measures BMI, body fat (%) and waist circumference were positively and significantly correlated to HOMA-IR, *P* < 0.001. More results about HOMA-IR are presented in the next paragraph.

### HOMA-IR

In the 809 subjects with values on HOMA-IR a recommended intake of fish and seafood (*P* < 0.05), fruit and vegetables (P < 0.001), and sweets (candy, buns, soft drinks and French fries) (P < 0.05) were associated to lower HOMA-IR values as compared to subjects not eating as recommended, see Table 4.

**Table 4:** The table shows HOMA-IR values for the groups “eating as recommended” and “not eating as recommended” for the seven food groups

Group	Food type	HOMA-IR	HOMA-IR	*P* value
		Subjects eating as recommended	Subjects not eating as recommended	
1	Bread	1.75 (*n* = 89)	1.82 (*n* = 717)	*P* = 0.553
**2**	**Fish and seafood**	**1.55** (*n* = 93)	**1.79** (*n* = 713)	***P*** **= 0.036**
**3**	**Fruit and vegetables**	**1.48** (*n* = 125)	**1.81** (*n* = 681)	***P*** **= 0.001**
4	Fat 1.75 (*n* = 247)		1.78 (*n* = 542)	*P* = 0.636
5	Cheese (20–40%)	1.77 (*n* = 304)	1.76 (*n* = 502)	*P* = 0.274
	**Sweets**	**1.64** (*n* = 330)	**1.85** (*n* = 476)	***P*** **= 0.039**
7	Fast food	1.75 (*n* = 722)	1.88 (*n* = 84)	*P* = 0.246

Clarification: The group named “sweets” includes candy, buns, soft drinks and French fries

Significance level was set to *P* < 0.05, significant *P* values are marked bold

**Fig. 1:**
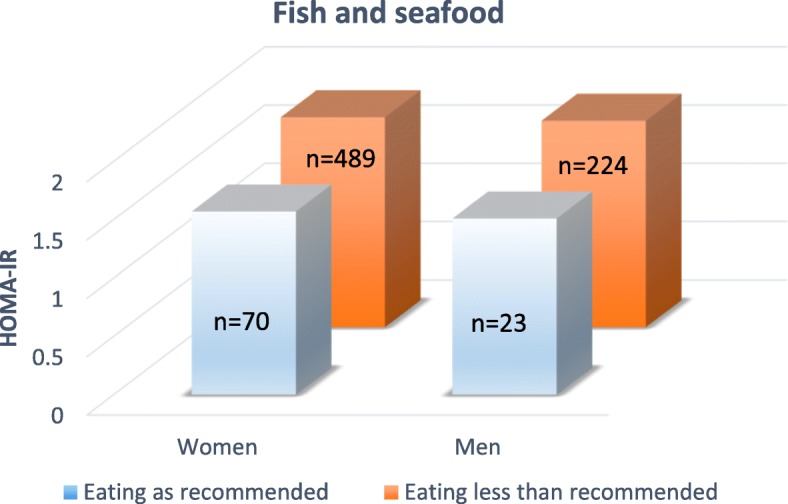
Insulin resistance (HOMA-IR) compared between subjects eating fish and seafood as recommended, with subjects eating less fish and seafood than recommended. According to the Swedish national food administration fish or seafood should be eaten as main course two times per week or more. Comparisons between the two groups have been made in women (*n* = 559) and men (*n* = 247) separately. Significance level was set to *P* < 0.05

When splitting the groups by sex, the difference in HOMA-IR with a recommended intake of fish and seafood, did not remain. Of the women 12.5% and of the men 9.3% was eating fish and seafood as recommended, see Fig. 1.

**Fig. 2:**
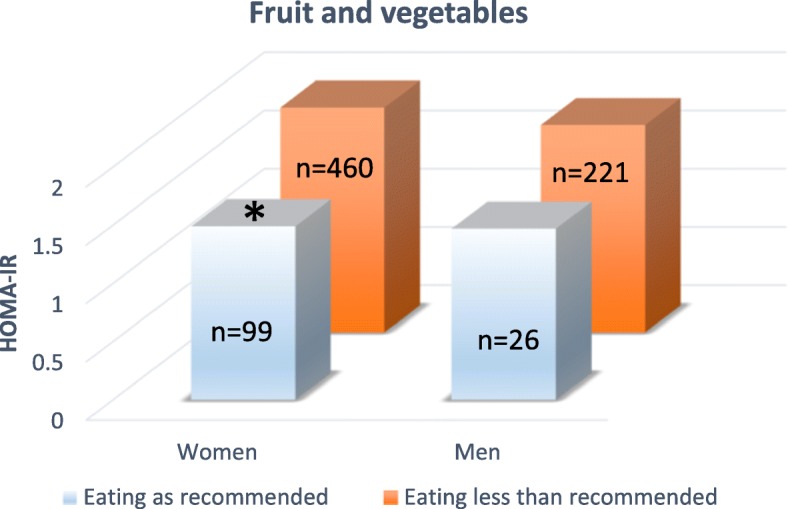
Insulin resistance (HOMA-IR) compared between subjects eating fruit and vegetables as recommended, with subjects eating less fruit and vegetables than recommended. The recommended intake, according to the Swedish national food administration, is 500 g of fruit and vegetables per day or more. Comparisons between the two groups have been made in women (*n* = 559) and men (*n* = 247) separately. Significance level was set to *P* < 0.05

Women with a recommended intake of fruit and vegetables had significantly lower HOMA-IR values (*P* < 0.01), compared to women eating less fruit and vegetables, but no difference was detected in men. Of the women 17.7% and of the men 10.5% was eating fruit and vegetables as recommended, see Fig. 2.

**Fig. 3:**
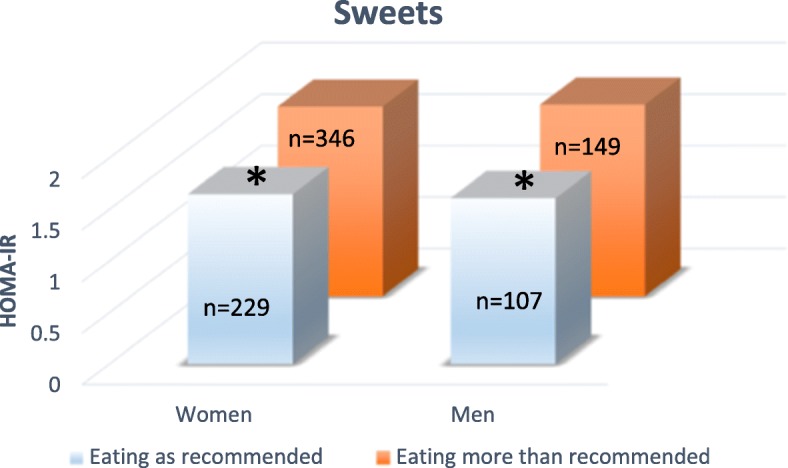
Insulin resistance (HOMA-IR) compared between subjects eating sweets i.e. on buns, candy, soft drinks and French fries as recommended, with subjects eating more sweets than recommended. According to the Swedish national food administration sweets should be eaten less than three times per week. Comparisons between the two groups have been made in women (*n* = 559) and men (*n* = 247) separately. Significance level was set to *P* < 0.05

Both women and men eating more sweets than recommended had significantly higher HOMA-IR values, women (*P* < 0.05) and men (*P* < 0.05), compared to subjects following the recommendations from the Swedish national food administration regarding candy, buns, soft drinks and French fries. Of the women 39.9% and of the men 43.3% was eating sweets as recommended, see Fig. 3.

## Discussion

In the Lifestyle, Biomarkers and Atherosclerosis (LBA) study we have previously reported that the high prevalence (15%) of Swedish, young adults with unfavorable homeostasis model assessment of insulin resistance (HOMA-IR) raises concerns about future health [7]. The main findings from the present study were that subjects with a high intake of food rich in sweets had higher insulin resistance (HOMA-IR) values as compared to subjects eating less sweets. Young adults with a sufficient intake of fish and seafood on the other hand had lower HOMA-IR values compared to subjects not eating fish and seafood as recommended, and subjects with a recommended intake of fruit and vegetables had significantly lower HOMA-IR values and less body fat (%) as compared to subjects eating less fruit and vegetables.

Arts et al. have studied CVD risk in college students, 18–24 years, in the USA. Similarly to us Arts et al. have found a high prevalence of CVD risk factors. They highlight the importance of screening young adults for CVD risk, with regard to lifestyle factors such as dietary habits [21]. Most of young adults are unaware of their risk and are ideal targets for prevention since they are in the process of establishing lifestyle habits. Risk factors in the young adulthood strongly predict CVD risk. It is known that reaching middle age free from traditional CVD risk factors are associated with longer life expectancy, and lower risk of developing CVD later in life [21].

### Body composition

As mentioned in the introduction Fernandes et al. have examined the influence of dietary habits on CHD risk in College students in the USA. They found that healthy dietary habits with sufficient intake of whole grain and reduced intake of sugar were associated with reduced CHD risk. They conclude that dietary habits and BMI are important indicators of CHD risk [12]. In the present study no association of healthy dietary habits to the body composition measures BMI or waist circumference was found in any of the seven examined food groups.

However, body fat (%) was significantly lower in women and men eating fruit and vegetables five times per day or more which means that they reached the recommended intake of 500 g per day. Equally, lower body fat (%) was seen in woman eating fish and seafood as recommended. The results indicate that body fat (%) is also a sensitive biomarker in this age group. Body fat distribution and adipose tissue dysfunction are key factors in the development of CVD and obesity-related insulin resistance, and better predictors of disease risk, compared to BMI and body fat (%) [22]. However, BMI and body fat (%) are both important determinants of metabolic health at the population level. The present study aimed to associate food habits to metabolic health at population level. A limitation, except for the lack of measurement on body fat distribution, was that a majority of the young subjects were women, see Table 1.

Reduced intake of food choices rich in sugar might in the long run reduce weight, and thereby contribute to a healthy aging process. Both women, and men with a higher than recommended consumption of sweets had higher HOMA-IR values but at this age no difference in BMI, body fat (%) or waist circumference as compared to subjects eating less sweets. The result was surprising to us but is supported by a number of epidemiological studies showing that sweets do not contribute much to body weight gain in normal-weight individuals [23].

In Sweden media have been supportive for a low-carbohydrate-high–fat (LCHF) diet and intake of carbohydrates have decreased in both genders [4]. A possible explanation for the low intake of bread (whole grain) and fruit observed in the present study may be changes in dietary habits, with less carbohydrate, and that fruit is perceived as an alternative rich in carbohydrates and sugar. In the present study on young adults only 6.9% answered that they were following a LCHF like diet and the majority of young adult’s participating in the LBA study chose unsaturated fat in cooking. The majority (89.2%) of the subjects were also following the recommendations on fast food and sausage.

### HOMA-IR

The recommendations on whole grain and fish and seafood were hardest for the subjects to follow. Most of the subjects (88.6%) in the present study did not eat enough of fish and seafood to reach the recommendations. In the present study a recommended intake of fish and seafood was significantly related to healthy insulin resistance (HOMA-IR) values when women, and men were analyzed together. When split by sex no significance was reached. The lack of significance was most likely due to the low amount of subjects eating fish and seafood as recommended (11.4%), see Table 4 and Fig. 1.

In the present study the majority of the subjects had lower than recommended intake of fruit and vegetables. When women and men were analyzed together a recommended intake of fruit and vegetables were related to lower insulin resistance (HOMA-IR) values. But, when split by sex a significant difference was observed only in women. The lower number of men participating in the study, in combination with the low number of participants eating fruit and vegetables as recommended (15.4%), was most likely the reason for the lack of significance in the male subjects, see Table 4 and Fig. 2.

The result is supported by several studies. In a large study, with results from different parts of the world, the importance of fruit and vegetable consumption, in relation to insulin resistance and diabetes have been pointed out [24]. Another example is a prospective Chinese study, 0.5 million Chinese adults were followed during 7 years to study the effect of fruit consumption on diabetes [25]. In China, and in many parts of the world, the relative high sugar content in fresh fruit have led to diminished fruit consumption in diabetic subjects. The findings of the Chinese study suggest that fresh fruit is beneficial for primary and secondary prevention of diabetes [25]. In a large study from Brazil, it was shown that among the dietary factors they studied a low intake of fruit, (< 300 g/day), was the largest contributor to cardiometabolic deaths. Next to fruit a low intake of whole grain and a high intake of sodium contributed most to cardiometabolic deaths [26]. In another Brazilian study on 4202 young adults, average age 23 years, a traditional Brazilian diet was compared to a diet based on processed food. The traditional diet showed generally healthier trends regarding CVD risk factors [27].

In Sweden, the importance of an adequate intake of fruit and vegetables are stressed by the Swedish national food administration [15]. It is also stressed in the Lifestyle recommendations for the prevention and management of metabolic syndrome [28].

The relation between high intake of fruit and vegetables, and HOMA-IR highlights the importance of increasing the intake of fruit and vegetables, with natural sweetening, fibers, vitamins and minerals, to prevent insulin resistance in young adults. It also highlights the importance of reducing the intake of sweet dietary choices, such as buns, candy and soft drinks.

The association of vegetable and fruit consumption with metabolic syndrome has recently been shown in a meta-analysis. A total of twenty-six observational studies were included and the evidence suggest that fruit and vegetable consumption is negatively associated with metabolic syndrome [29]. There are several articles on the topic and in another review 11 studies were included. All reported cross-sectional data. The majority of participants, with type 2 diabetes, were consuming less than the recommended amount of fruit and vegetables [30].

In the United States young adults have a high intake of sugar and saturated fat. The high intake of fat and sugar is aggravated by inadequate intake of fruit, vegetables and fibers [21]. More than half of the young American adults have at least 1 CHD risk factor. This greatly increases lifetime CHD risk [21]. In the present study the intake of sweets was high, only 40.1% of the participants were following the recommendations. Women and men following the recommendations had lower HOMA-IR values compared to women and men not following the recommendations on sweets, see Fig. 3. Unhealthy HOMA-IR values due to high intake of sweets were, as previously mentioned not reflected by increased body composition measures. BMI, body fat (%) and waist circumference were similar in subjects eating sweets as recommended and in subjects eating more sweets than recommended.

In the present study, only 11.4% of the participants had a sufficient intake of whole grain, but intake of whole grain was not associated to body composition measures or HOMA-IR values. Sufficient intake of whole grain gives an increased sense of saturation, and may thereby reduce snacking, and in the long run contribute to weight maintenance. It has been shown that whole grain reduces the risk of deteriorating glucose tolerance, including progression to prediabetes [31].

Lifestyle, and dietary habits have changed in Sweden [4] and worldwide [32]. A study on University students in Spain, similarly to the present study, shows that the majority of students, 96.1%, were recommended to improve the quality of their diets. The main deviations from a recommended diet, in the Spain University students, were a low intake of fruit and vegetables with a high intake of meat and diary products [33]. The observed association between less body fat (%) and lower insulin resistance (HOMA-IR) values in the present study, with dietary choices containing sufficient amounts of fish, seafood, fruit and vegetables gives support to the health beneficial effects of lifestyle interventions also in young adults [13].

### Strengths and limitations

It is important to study dietary habits and the risk of diabetes. The strength of the study lies in the study population, young adults. Limitations are that it was hard to recruit men, and therefore the majority of participants were women. In addition, many of the participants studied medicine and had an interested in health, which may have affected the result. Finally, most of the participants studied at Örebro University. We believe that Örebro University, located in the middle of Sweden, reflects the Swedish University’s, but regional differences cannot be ruled out.

## Conclusions

In the LBA study we have previously noticed a high prevalence of insulin resistance (HOMA-IR) in Swedish, young adults. In the present study we have additionally observed that the adherence to the recommendations on healthy food choices from the Swedish national food administration are low, with consequences on metabolic risk markers. Women and men eating more sweets than recommended had higher HOMA-IR values as compared to subjects not eating as much sweets. Sufficient intake of fish and seafood on the other hand was associated to lower HOMA-IR values compared to subjects not eating fish and seafood as recommended. Women and men with sufficient intake of fruit and vegetables had significantly lower HOMA-IR values and less body fat (%). The result highlights the importance of reducing a high intake of sweets and to increase the consumption of fish, fruit and vegetables, in young adults, to reduce the risk of future diabetes.

## Abbreviations

AFA: Asset Management Arm
BMI: Body mass index
CHD: Coronary heart disease
CVD: Cardiovascular diseases
HDL-C: High-density lipoprotein cholesterol
HOMA-IR: Homeostasis model assessment of insulin resistance
hs-CRP: High-sensitive C-reactive protein
LBA study: Lifestyle, Biomarkers, and Atherosclerosis study
LCHF: Low-carbohydrate-high-fat
STD: Standard deviation

## Author’s information

Maria Fernström: Åstrand Laboratory of Work Physiology, The Swedish school of sport and health science, GIH, Lidingövägen, 1, 114 86 Stockholm, Sweden. maria.fernstrom@gih.se

Ulrika Fernberg: School of Medical Sciences, Faculty of Medicine and Health, Örebro University, 701 82 Örebro, Sweden. ulrika.fernberg@oru.se

Anita Hurtig-Wennlöf: School of Health Sciences, Faculty of Medicine and Health, Örebro University, 701 82 Örebro, Sweden. anita.hurtig-wennlof@oru.se
